# Medicare Part D plan-selection experience: qualitative findings from a national cross-sectional survey

**DOI:** 10.1016/j.rcsop.2022.100219

**Published:** 2023-01-03

**Authors:** Logan T. Murry, Matthew J. Witry, Julie Urmie

**Affiliations:** The University of Iowa College of Pharmacy, 180 S Grand Ave, Iowa City, IA 52358, United States

**Keywords:** Medicare part D, Patient experience, Inflation reduction act, Medication costs, Insurance, Pharmacy

## Abstract

**Background:**

A variety of services exit to assist eligible beneficiaries select Medicare Part D insurance plans; however, selecting an optimal plan remains a challenge. While patients would benefit from evaluating and switching their Medicare Part D plan on a yearly basis, few choose to do so.

**Objective:**

The objective of this study was to describe the Medicare Part D plan selection experience across all US states.

**Methods:**

This was a qualitative analysis using data from a cross-sectional Qualtrics panel survey administered in January 2022. Descriptive statistics were generated for demographic and patient-specific items for individuals who provided open-ended survey item responses. Open-coding and content analysis were used to analyze responses to the open-ended survey item.

**Results:**

Overall, 540 responses were received, with the largest number of responses from Florida (11%, 61). A total of 101 respondents (18.7%) of survey respondents provided open-ended comments. Qualitative analysis identified four response categories: Benefit design, Plan information and selection assistance, Plan Switching, and Plan-selection experience.

**Conclusions:**

Overall, participants expressed frustrations with high costs and plan restrictions. Many participants needed plan-selection assistance, with some individuals switching plans each year. Recent legislation may address difficulties related to medication costs; however, additional focus on resources and educational interventions may improve the Medicare Part D experience.

## Highlights

• Participants had varied experiences with Medicare Part D plan selection.
• Participants expressed concerns with benefit design and medication costs, requiring varying degrees of assistance.
• Additional policy considerations may be required to address difficulties with the Medicare Part D insurance experience not addressed by the Inflation Reduction Act.

## 1 Introduction

The Medicare Part D insurance program began in the USA in 2006, providing Medicare beneficiaries with outpatient prescription medication coverage.^1^ While many individuals enroll in Medicare Part D insurance plans, a proportion of eligible beneficiaries have opted to enroll in Medicare Advantage plans, private insurance plans paid on a capitated basis by the Centers for Medicare and Medicaid Services (CMS).^2^^,^^3^ Enrollees rely on Medicare Part D and Medicare Advantage to afford prescription medications; however, the insurance selection processes continues to be a challenge for Medicare-eligible individuals. Many enrollees select suboptimal plans and remain in plans which may result in larger-than-necessary out-of-pocket expenditures.^4^^,^^5^

Suboptimal plan selection and plan inertia may be attributed to a variety of factors, including but not limited to: confusion caused by the large number of available plans,^6^ variations in plan benefit designs,^7^, ^8^, ^9^ and plan-selection requiring beneficiaries to navigate a large amount of complex health information effectively and efficiently, a process requiring high levels of self-efficacy, health activation, and health insurance literacy.^10^ Recognizing the ongoing difficulty with insurance plan selection, groups and organizations across the country have initiated interventions designed to provide Medicare insurance education, helping eligible beneficiaries with the plan-selection process, identifying lower cost plans and encouraging plan-switching.^11^ Medication costs with Medicare insurance coverage has received increased attention, with the Inflation Reduction Act passed to address rising medication costs for patients with Medicare insurance.^12^, ^13^, ^14^

Several studies have focused on evaluating patient experiences with Medicare Part D plan selection, community pharmacies providing plan-selection assistance, and benefits of these services at the patient level. A study by Stults et al. (2018) evaluating the patient experience in selecting a Medicare Part D plan found that identifying low-cost plans was essential to study participants; however, other characteristics such as convenience and company reputation were also important.^15^ Further, study participants reported using resources to assist in the plan-selection process and expressed a desire for more assistance and a simplified plan-selection process. A study by Murry, Al-Khatib, and Witry (2021) evaluated the patient experience of selecting a Medicare Part D plan for individuals who did and did not use a community pharmacy consultation service. The study identified that while pharmacy consultation services may help to inform and educate patients about the plan-selection process, the amount of information provided may make the experience more challenging.^16^ These studies were conducted with relatively small samples within two geographic locations (Northern California and Iowa). As such the objective of this study was to understand patient perceptions of the Medicare Part D plan-selection experience across the US.

## 2 Methods

A cross-sectional survey was developed using existing literature on Medicare Part D plan-selection experience and author expertise. Survey items collected information on respondent demographics (age, gender, education, area of residence, difficulty affording prescription medications, health activation, and annual household income), pharmacy and pharmacy services (pharmacy patronage, number of pharmacies used in the past 30 days, number of prescription medications, and pharmacy service use), and an open-ended item collecting responses surrounding Medicare Part D plan selection and experience. The open-ended item read as follows: “Finally, is there anything else you would like to share about your experiences selecting a Medicare Part D insurance plan or the idea of community pharmacies helping with Medicare Part D plan selection?”

The survey was initially pretested with six researchers at The University of Iowa College of Pharmacy with expertise in Medicare Part D, pharmacist-patient relationships, and survey design and analysis. During pretesting, content and face validity of items were assessed. The survey was then pretested with a purposeful convenience sample of 15 Medicare-enrolled participants. Pretesting resulted in the following changes to the survey instrument: the introductory material for survey was, all ranking questions using a slider for response selection were changed to inform individuals that the slider was how their response would be recorded, and multiple page breaks were inserted in the final block of the survey to minimize scrolling, presenting individuals with more pages with fewer questions on each page. The revised survey was administered via a national Qualtrics survey panel in January of 2022. Patients who had previously registered for survey participation through Qualtrics were contacted via electronic mail to participate in an electronic survey, with Qualtrics providing participants with a point-based incentive valued at approximately 20–40% of the survey cost ($1.85 to $3.70 USD). Inclusion criteria to participate in the survey was: English-speaking U.S adults (≥65 years) who were currently enrolled in a Medicare Part D or Medicare Advantage plan and had filled a prescription at a pharmacy within the last 12 months. The survey was piloted with a sample of 50 participants recruited by Qualtrics, prior to full survey administration. No prior relationship was established with participants, participants were informed study results would be used to improve Medicare consultation services, and study activities were reviewed and approved by The University of Iowa Institutional Review Board, IRB#202106181, in November of 2021.

A heat map was generated to depict all survey responses by state. For individuals who provided open-ended survey item responses, frequencies and descriptive statistics were generated. Additionally, survey responses for participants who did and did not complete the open-ended item were compared using Chi-square and Kruskal Wallis tests to assess variation between groups and potential response bias. Content analysis using a general interpretivist approach was performed on the open-ended response item.^17^ Open codes were originally assigned to text responses by one study author (LTM) using NVIVO (Version 12, 2020). The positionality of the original coder is a male pharmacist and PhD trained researcher with extensive training in qualitative analysis and expertise in patient experience with Medicare Part D insurance selection. A second study author (MJW) reviewed the coded text segments and the two authors met to discuss identified codes and potential categories to group quotes. After agreement was reached, a third study author (JU) with expertise in Medicare Part D insurance selection reviewed the codes and identified themes, with all authors reaching agreement on the finalized coding and content analysis. Accuracy and validity in qualitative analysis was promoted using several strategies, including investigator triangulation, presenting researcher reflexivity, and fairness and balance in the presentation of quotes.^18^ Representative quotes which reflected the participant voice were selected and included in the results. Study results were reported using the COREQ guidelines.^19^

## 3 Results

**Fig. 1:**
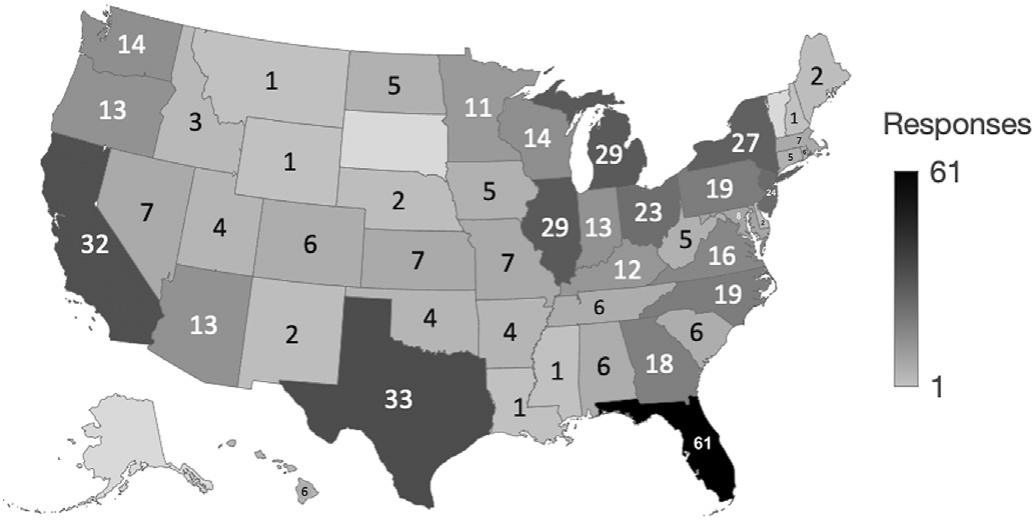
Heat map of survey responses for each US state (*n* = 540).

In total, 642 participants started the survey, with a total of 540 responses received for a response rate of 84.1%. Florida was the state with the largest number of responses (11%, 61) and responses were received from all states except Alaska, South Dakota, and Vermont. The total number of responses from each US state is visually depicted in Fig. 1.

A total of 101 survey respondents (18.7%) provided open ended comments. The mean ± standard deviation age of participants who responded to the open-ended item was 72.0 ± 5.8. The majority of participants who responded to the open-ended item were female (*n* = 64, 63.4%), used one pharmacy in the past 30 days (*n* = 81, 80.2%), were currently taking 4 or more prescription medications (*n* = 52, 51.5%), and were residents in a “Suburban” area (*n* = 57, 56.4%). Only 25 participants (24.8%) reported currently taking a prescription medication they found difficult to afford. For all respondents, the mean ± standard deviation for health activation was 7.5 ± 1.9. Frequencies and descriptive statistics for additional items not fully described in the text are included in Table 1. There was a statistically significant difference in health activation between individuals who did and did not provide a response to the open-ended item, with mean ± standard deviation health activation scores of 7.1 ± 2.1 and 7.6 ± 1.9, respectively. There were no other statistically significant differences in responses between groups.

**Table 1:** Participant demographics for open-ended survey responses (*n* = 101).

Item	Frequency (%)
Gender	Male =37 (36.6) Female = 64 (63.4)
Education	Some high school = 3 (3.0) High School or Graduate Equivalency Degree = 15 (14.9) Some College = 37 (36.6) Bachelor's degree or advanced graduate work = 46 (45.5)
Residence	Urban =14 (13.9) Suburban = 57 (56.4) Small Town =12 (11.9) Rural = 18 (17.8)
Difficulty Affording Prescription Medication	Yes = 25 (24.8) No = 76 (75.3)
Annual Household Income	Under $25,000 = 21 (20.8) $25,000 to $49,000 = 34 (33.7) $50,000 to $74,999 = 19 (18.8) $75,000 or more = 27 (26.7)
Pharmacy Patronage	Chain = 38 (37.6) Independent = 9 (8.9) Grocery = 9 (8.9) Mass Merchandiser = 14 (13.9) Multiple = 31 (30.7)
Different Pharmacies Used in the Last 30 days	1 = 81 (80.2) 2 = 16 (15.8) 3 or more = 4 (4.0)
Prescriptions Currently Taking	1 = 14 (13.9) 2 = 11 (10.9) 3 = 24 (23.8) 4 or more = 52 (51.5)
Previous Pharmacy Service Use	Yes = 65 (64.4) No = 36 (35.6)

### 3.1 Qualitative responses

Content analysis identified four response categories across all respondents: *Benefit Design, Plan information and Selection Assistance, Plan Switching,* and *Plan-Selection Experience.* Themes and codes can be found in Table 2, with text reflective of each code and state from which the response was collected included. The following text describes the codes.

**Table 2:** Qualitative response categories, exemplary quotes, state, and codes.

Category	Representative Text (State)[Code]
Benefit Design	*I have to pay for a drug plan even when I don't use it. I have to use GoodRx (KY) [Cost]* *One of my prescriptions is less expensive paying direct rather than using my insurance.* (RI) *[Cost]* *The cost is outrageous for a single individual with one prescription. Pharmaceutical companies should be restricted to what they can charge for prescription medications, It's ludicrous how much medicine costs and it's not fair to those without insurance. (CA) [Cost]* *Do not like the restriction on use of manufacture coupon offers to help cover copay when Plan D is being use. (NJ) [Plan Restrictions]* *I wish my coverage allowed me to use the pharmacy of my choice because it would not be [PHARMACY]. (KY) [Plan Restrictions]*
Plan Information and Selection Assistance	*Too many advertisements, and none are from**Medicare.gov**. (GA) [Information]* *I get too many letters in the mail and it all makes it very hard to choose any of them. I mostly think this whole thing is a major scam. (NC) [Information]* *I would prefer a government plan instead of so many different options (CA)[Information]* Any help is appreciated especially if it helps lower costs (AZ) *[Plan-selection Help]* *I feel that an insurance specialist would be better at providing good options. I don't want the pharmacy bogged down by providing information that is better provided by an experienced insurance agent. (NV) [Insurance Agent]* *We are going to talk to an employee with our primary care doctor to see what is best for us, in the near future. (PA) [Health Care Provider]* *I rely primarily on**Medicare.gov**when selecting my health coverage each year. My local pharmacies are usually too busy to help me with this. (MN) [Medicare Website]* *I only think it is an excellent idea to have pharmacy help in a community where one actually lives in selecting their drug plan. (FL) [Pharmacy]* *With volume of customers [PHARMACY] has, think this plan would impact ability to do their actual job of filing and advising on prescriptions (CA) [Pharmacy]*
Plan Switching	*I think it's good, I just keep blindly re-enrolling an maybe there are better options. (IL) [Plan Inertia]* *No, I stick with the same plan I picked when I turned 65 (NJ) [Plan Inertia]* *I changed insurance to help pay for medications. (NY) [Change Plan]*
Plan-selection Experience	*All the calls trying to sell Part D plans. Don't know who to trust. Very confusing. (WY) [Confusing]* *Choosing a plan can be difficult for seniors. It's overwhelming. (UT) [Overwhelming]* *Feel process is more complicated than it needs to be. (MI) [Complicated]* *Just want to make sure [I'm] on the best plan. (TN) [Uncertainty]* *It is confusing for most seniors and to top it off prices tend to move up and down more than the stock market! (PA) [Frustration and Confusion]* *I had no problems selecting the Medicare Part D that was perfect for me. (MN) [No Difficulty in Plan Selection]*

Participants expressed concerns with *Benefit Design*, most notably costs of medications and insurance plans, noting that prescriptions were less expensive if they bypassed their insurance or used programs like GoodRx. Other participants emphasized that plan restrictions on pharmacy use and the inability to use coupons were frustrating components of their Medicare Part D experience.

Participants highlighted various components of *Plan Information and Selection Assistance,* suggesting that too much information and advertisement came from insurance companies, with little information from Medicare.gov. One participant suggested that they would prefer a government plan rather than multiple different options. The majority of respondents reported using insurance agents or health care providers] to make the process less difficult. A participant reported that an insurance specialist might be more equipped to provide assistance, while another expressed concerns with a pharmacist-provided service, as staffing and workflow might be affected with pharmacies too busy to offer this service. A number of participants had positive comments about how their pharmacy could provide Medicare Part D plan selection assistance. A number of patients reported using the Medicare website to make their plan selection with little difficulty.

For *Plan Switching*, one participant continued to “blindly” re-enroll in their current plan, acknowledging better options may exist. Another participant reported staying in the same plan since becoming Medicare eligible at 65 years of age, while one participant mentioned switching plans to better cover the costs of their medications.

When considering the *Plan-Selection Experience*, participants reported frustration, confusion, uncertainty, and feeling overwhelmed. While participants reported struggling with selecting a plan, two participants located in the Midwest reported no difficulty with plan selection. Most individuals focused on the number of plans available, how medication costs vary across plans and change on a yearly basis, and difficulties with identifying and choosing the best plan.

## 4 Discussion

Overall, participants in this study had a wide variety of experiences surrounding Medicare Part D plan selection with comments related to costs, plan information, and plan selection. From content analysis, four categories of responses were identified: benefit design, plan information and selection assistance, plan-selection experience, and plan inertia. Themes had a degree of overlap, with benefit design, plan information and selection assistance, and plan inertia contributing to the plan-selection experience. Patients with benefit design concerns and lack of plan information and plan-selection assistance appeared to have more negative experiences, while those who were able to identify and obtain assistance, independently use existing resources (i.e., Medicare.gov), or did not consider switching (e.g., plan inertia) seemed to have more positive experiences.

Across the US, the benefit design element of cost was frequently a consideration for participants when selecting their Medicare plan. While many individuals were more generally concerned about cost, others had specific frustrations with their plan benefits and structure, including restriction on manufacturer coupons, pharmacy restrictions, bypassing insurance to decrease out-of-pocket costs, and frustrations with medication and plan cost or coverage. These findings are similar to those in the existing literature, as plan and medication costs have frequently been noted as important considerations for Medicare plan selection.^15^^,^^16^^,^^20^ These concerns may also reflect continued changes in Medicare plan-benefit structure, with the monthly premiums for some Medicare Part D plans continuing to rise year-over-year and out-of-pocket medication costs may remain burdensome as a result of co-payments and deductibles despite Medicare Part D insurance coverage.^13^ Policy recommendations provided by the American College of Physicians address patient difficulties, concerns, and frustrations related to costs associated with benefit design. They support annual out-of-pocket spending caps for Medicare Part D beneficiaries who reach the catastrophic phase of coverage, which would potentially address the concerns of multiple patients expressing frustration with medication cost despite insurance coverage.^21^ Cost-related concerns from Medicare Part D patients and recommendations from the American College of Physicians may partially be addressed by the Inflation Reduction Act, which became U.S. law in 2022. This law includes caps on out-of-pocket prescription medication maximums for Medicare beneficiaries, allows Medicare to negotiate the prices of 100 drugs, and requires drug companies to provide rebates to Medicare for price increases higher than inflation.^14^^,^^22^^,^^23^

Participants in this study also reported difficulties navigating Medicare Part D plan information, with participants noting the volume and variety of marketing material they were exposed to. In Contract Year 2019, the Centers for Medicare and Medicaid Services (CMS) updated the definition of marketing, altering requirements surrounding materials which would be subject to CMS review while potentially decreasing CMS oversight on marketing and plan information materials provided by plan sponsors.^24^ This may have resulted in an increase in the volume and frequency of plan marketing and information exposure. For Contract Year 2023, CMS reversed course, increasing oversight of marketing and communication, which may improve patient access to and navigation of information moving forward.^25^

Additionally, patients may have limited access to Medicare selection assistance, infrequently use existing services, or have suboptimal experiences with existing service offerings. Budget cuts have affected State Health Insurance Assistance Programs (SHIPs), which provide information and counseling for Medicare Part D plan selection, potentially decreasing access to Medicare Part D plan selection assistance.^26^ Although recent increases to the Medicare budget and improvements to the Medicare.gov website may improve access to plan selection assistance, the plan-comparison and selection experience continues to be a challenge.^26^, 27., 28., 29. To address these concerns, resources could be allocated to Medicare Part D education resources to improve access to Medicare Part D plan information. Additionally, CMS regulations on marketing and information for Contract Year 2023 could be evaluated for effect and intended outcomes. Additional policies could be considered to improve plan information and marketing materials presented to patients, in addition to supporting access to Medicare Part D plan information and plan-selection assistance.

For plan selection experience, assistance, and inertia, policies and resources could again focus on existing Medicare Part D education resources. Plan inertia is common in existing Medicare Part D plan selection literature, with few patients choosing change plans despite potentially less expensive plan alternatives.^10^^,^^30^, ^31^, ^32^, ^33^ Community pharmacy-led Medicare Part D consultation services have shown to mitigate the effects of plan inertia, helping patients switch to potentially more appropriate Medicare Part D plans while improving chronic medication adherence.^30^ Despite these benefits, plan-selection assistance may negatively affect the plan-selection experience, as assistance may increase exposure to plan information and increase patient perceptions of pressure to switch plans.^16^ Additional policy could be explored to help provide remuneration and necessary support for groups and organizations providing Medicare Part D education to develop sustainable interventions. Groups and organizations could also consider ways to optimize service delivery and patient experience with Medicare Part D plan selection services.

## 5 Limitations

The study has a number of limitations. Some states only had one respondent, limiting the ability to make state-specific conclusions. The small number of open-ended responses compared to total survey responses has the potential to introduce respondent bias, with only polarizing experiences captured. Further, data collection via open-ended item potentially limits the ability to determine true data saturation; however, the number of responses and responses collected from 47 US states suggests an adequate amount of data has been collected to identify categories. Additionally, individuals who left open-ended responses had significantly lower levels of health activation compared to those who did not leave a response, potentially capturing open-ended responses from a group of individuals requiring more support related to health decisions. Further, the population of this study appeared to have completed a higher level of education and were more likely to be female compared to 2019 Medicare population,^34^^,^^35^ which may impact the generalizability of these results to the larger Medicare population. Future research could consider expanding on these findings, using alternative data collection methods such as interviews or focus groups to explore Medicare-eligible participant experience across the US. Additionally, future work could explore limitations in this study, specifically testing the effect of health activation and education on the Medicare plan-selection experience.

## 6 Conclusions

Overall, patients reported a wide variety of Medicare Part D plan selection experiences, with many individuals focusing on the cost of their plans, the amount of information they received, and the necessity of a helper to make the plan decision. While the Inflation Reduction Act may curb concerns related to medication cost, it may not alleviate other difficulties associated with Medicare Part D plan selection such as plan inertia, the number of available plan providers actively soliciting patients, and the confusion and uncertainty surrounding available plan options. Additional policies, resources, and support are needed to improve the Medicare Part D insurance experience for many individuals across the US.

## Funding

This work was supported by the Iowa Pharmacy Association and the Outcomes Innovative Pharmacy Grant Program.

## Declaration of Competing Interest

The authors have no conflicts of interest.
